# Identification, functional analysis, and clinical applications of defective viral genomes

**DOI:** 10.3389/fmicb.2025.1642520

**Published:** 2025-07-17

**Authors:** Xiaowei Yan, Yitong Pan, Peiying Li, Li Zhu, Jianhai Yu, Chenguang Shen, Bao Zhang, Wei Zhao

**Affiliations:** ^1^BSL-3 Laboratory (Guangdong), Guangdong Provincial Key Laboratory of Tropical Disease Research, Ministry of Education Key Laboratory of Infectious Diseases Research in South China, School of Public Health, Southern Medical University, Guangzhou, China; ^2^School of Biomedical Engineering, Southern Medical University, Guangzhou, China

**Keywords:** interfering particle, defective viral genome, identification, function, application

## Abstract

Defective viral genomes (DVGs) are fragments derived from defective interfering particles (DIPs) that form during viral replication. They play important roles by interfering with complete virus replication and regulating host immune responses. Advances in high-throughput sequencing (HTS) and bioinformatic technology have significantly improved the ability to identify DIPs and DVGs. Their heterogeneity and dynamic formation mechanisms have been analyzed using long-read sequencing technologies. Both DIPs and DVGs inhibit wild-type viral proliferation by competing for viral replication resources and activating innate immune pathways such as those of retinoic acid-inducible gene 1 and mitochondrial antiviral signaling protein. This might influence infection outcomes by regulating inflammatory cytokine storms. The clinical application of DIPs and DVGs in their natural attenuated virus forms has been investigated in terms of novel vaccine design and antiviral therapy. This report systematically reviews cutting-edge detection techniques, molecular mechanisms, and translational medicine advances of DIPs and DVGs and provides a theoretical basis for developing broad-spectrum antiviral strategies based on DIPs.

## 1 Introduction

Infectious diseases pose a substantial global threat to human health and wellbeing (Kirtane et al., 2021), with each outbreak or pandemic having significant implications for public health systems and societal stability (Murphy, 2022). The diagnosis and management of viral infections warrant significant attention because they are primary causative agents of infectious diseases (Gebreyes et al., 2014). The severe acute respiratory syndrome coronavirus 2 (SARS-CoV-2) Coronavirus 2019 (COVID-19) global pandemic lasted for almost 3 years, and resulted in >760 million confirmed infections and 6.9 million deaths (Yuan et al., 2023). The actual figures are believed to be significantly higher. Other viruses such as influenza (Taubenberger and Kash, 2010), Ebola (Jacob et al., 2020), dengue (Martina et al., 2009), and hepatitis B viruses (Yuen, 2018), continue to pose significant threats to human health. Developing effective vaccines and therapeutic agents is considered the most promising strategy for controlling infectious diseases (McArthur, 2019). However, the inherent biological characteristics of viruses pose significant challenges that complicate efforts to combat viral infections (Horvath and Barrangou, 2010), emphasizing the need for more effective treatments.

**Figure 1:**
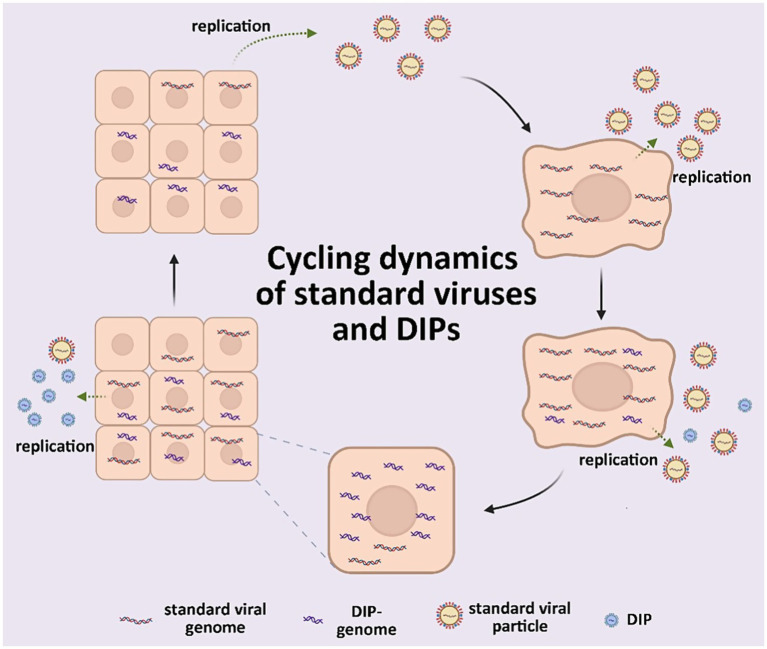
Scheme of standard viruses and DIPs cycling dynamics.

Numerous defective virus particles with biological activity identified in animal viruses during 1970 led to the concept of defective interfering particles (DIPs) (Huang and Baltimore, 1970). These particles are typically viral structural proteins that contain a segment of a viral genome and can replicate in the presence of a helper virus. They specifically inhibit the intracellular replication of unimpaired, homologous viruses. Defective viral genomes (DVGs) are truncated versions of their parent genomes that arise from an abnormal cycle of viral genomic replication (Brennan and Sun, 2024). Standard viruses (Genoyer and López, 2019) have complete genomes, which enables them to independently undergo an entire replication cycle unlike DIPs. Helper viruses (Manzoni and López, 2018) facilitate the replication and other functions of DIPs. The functions of DIPs include reducing virulence *in vivo* (Barrett and Dimmock, 1984; Rabinowitz and Huprikar, 1979), inducing robust interferon (IFN) expression during infection *in vitro* (Fuller and Marcus, 1980; Johnston, 1981), and promoting enhanced viral persistence *in vivo* (Baczko et al., 1986) and *in vitro* (De and Nayak, 1980; Kawai et al., 1975; Roux and Waldvogel, 1981; Schmaljohn and Blair, 1977; Sekellick and Marcus, 1978) (Figure 1). Despite potent functionality, DIPs and their associated DVGs were initially considered as artifacts of viral replication *in vitro* that were unrelated to natural viral infections, owing to technological limitations that prevented their identification *in vivo* (Viola et al., 1978). Thus, investigation into DIPs was constrained by such limitations and gradually stagnated for decades.

However, recent scientific and technological advancements, particularly the advent of second-generation sequencing technologies, have led to the discovery of DVGs among many viral families (Pathak and Nagy, 2009; López, 2014; Rezelj et al., 2018). Concurrently, DVGs derived from RNA viruses modulate immune responses to viral infections, indicating their potentially crucial role in determining infection outcomes (Genoyer and López, 2019). This review examines the research technologies, mechanisms of action, and clinical applications of DIPs and their associated DVGs, to elucidate their roles in viral infection and facilitate their eventual clinical applications.

The infection process begins when a cell is infected by the standard virus, which then disseminates to adjacent cells. In the presence of the standard virus, DIPs undergo conditional replication, thereby disrupting the replication cycle of the standard virus and promoting the accumulation of DIPs while effectively reducing the viral titer of the standard virus.

## 2 Technical support

Current identification and characterization of defective virus genome sequences primarily depend on high throughput sequencing (HTS; also known as next-generation sequencing, NGS) technologies and bioinformatic analyses. This report elaborates on the application of these methods to the study of DVGs.

### 2.1 Application of HTS technologies in viral genome sequencing

High throughput sequencing can simultaneously analyze vast numbers of DNA or RNA molecules within a very brief timeframe. This technology has been extensively applied to genomics, transcriptomics, and epigenetics, including the critical task of determining viral genome sequences (Slatko et al., 2018; Sharma et al., 2018).

Plaque assays in susceptible cell cultures are widely used to detect and quantify most viral species. However, conventional plaque assays cannot reliably quantify DVGs, as they do not complete replication cycles (Muñoz-Sánchez et al., 2024; Marx, 2023). The isolation and quantitation of DVGs using biochemical techniques have posed significant challenges. Consequently, the primary approaches to DVGs have shifted towards sequencing technologies and bioinformatics methodologies (Hwang et al., 2018).

Compared with conventional sequencing techniques, HTS simultaneously analyses a vast number of DNA or RNA fragments. This facilitates the rapid detection of DVGs in samples (Trebbien et al., 2018; Sheng et al., 2018) and significantly enhances sequencing efficiency. Comprehensive sequencing of the entire genome at multiple locations can be achieved in a single run through HTS, which also provides improved depth and exact mapping of DVG sites. The components of DVG have been analyzed in detail because of HTS technology. Advances in HTS technology have facilitated breakthroughs in elucidating the production (Alnaji et al., 2021; Pelz et al., 2021) and composition (Muñoz-Sánchez et al., 2024) of DVGs, which has significantly enhanced understanding of the various types of DVGs (Genoyer and López, 2019). Previous investigations into DVGs were frequently small-scale or constrained to specific viral species due to the prohibitively high cost of sequencing. The advent of HTS technology has significantly lowered these costs, facilitating a transition in DVG research from isolated case studies to comprehensive, large-scale explorations. It is noteworthy that in some reports, viral samples were amplified *in vitro* prior to HTS, leading to alterations in the population of defective viral genomes and consequently affecting sequencing results (Li et al., 2024). Although certain challenges remain, the advancement of HTS permits extensive sequencing analyses of diverse viral types and deeper investigations into the variability of DVGs.

### 2.2 Application of bioinformatics tools in identifying DVGs

#### 2.2.1 Strategies and algorithms used to identify DVGs

A popular strategy for DVG identification using HTS involves read alignment to a reference viral genome, followed by the localization of base-pair and relative index positions. Several bioinformatics tools have recently been developed in response to this need (Table 1). An early program called Paparazzi, was initially designed to exclude rather than to identify DVGs in samples for viral genome reconstruction (Vodovar et al., 2011). The first algorithm specifically designed for DVG identification in HTS data was ViReMa-a. Along with DI-tector, it remains the most widely used algorithm for DVG detection (Routh and Johnson, 2014; Beauclair et al., 2018).

**Table 1:** A comparison of legacy pipeline and next-generation pipeline.

Aspect	Legacy pipeline	Next-generation pipeline
Data source	Short-read sequencing [e.g., Illumina MiSeq (Wagner et al., 2025)]	Long-read sequencing [e.g., Nanopore (Ndekezi et al., 2025], PacBio [Jia et al., 2024)], hybrid sequencing
Detection method	Alignment-based detection [e.g., BWA (Bian et al., 2025), GATK (Wang et al., 2024)], simple deletions detection	Machine learning [e.g., DVGfinder (Olmo-Uceda et al., 2022)], structural variant calling [e.g., Sniffles (Sedlazeck et al., 2018)]
Tool dependency	Traditional aligners [e.g., Bowtie2 (Zhou et al., 2024), BWA], custom Perl or Python scripts	Specialized DVG tools [e.g., ViReMa (Sotcheff et al., 2023), DI-tector (Beauclair et al., 2018), DVGfinder]
Compute architecture	Single-node or basic high performance computing	Distributed computing
Sensitivity	Low (alignment-dependent, misses complex DVGs)	High (hybrid assembly and alignment, detects chimeric or rearranged DVGs)
False positive control	Manual filtering [e.g., IGV inspection (Robinson et al., 2023)]	Automated filters (ML models, statistical significance)
Standardization	Lab-specific, no consensus	Standardized workflows [e.g., ViReflow (Moshiri et al., 2022)]
Reproducibility	Low	High
Virus compatibility	Primarily RNA viruses	Both DNA and RNA viruses
Representative tools	Custom scripts, early ViReMa	ViReMa, DVGfinder, DI-tector, iVar (Castellano et al., 2021)

The machine learning (ML)-based, metasearch tool DVG-finder aimed to specifically and accurately identify DVGs in RNA-sequencing data (Olmo-Uceda et al., 2022). However, several algorithms, such as ViReMa-a and DI-tector, introduce bioinformatic artifacts during identification that could potentially lead to false positives or false negatives that reduce sensitivity (Bosma et al., 2019). By integrating ViReMa-a and DI-tector, DVG-finder has standardized terminology and incorporates additional descriptive variables into a unified workflow. This approach leverages ML to minimize false positives and provides an HTML report with graphical outputs, while preserving the distinct data generated by ViReMa-a and DI-tector.

#### 2.2.2 Operation mode and performance analysis of DVG-finder

The computational algorithms ViReMa-a and DI-tector are integrated into DVG-finder to detect DVGs in samples. Detected DVGs are processed through the distinct operating modes of meta-search, consensus, and filtering. All identified DVG data are shown in meta-search mode, whereas only DVGs detected by both algorithms are revealed in consensus mode. The filtering mode shows DVG data that meet or surpass a user-specified true-positive probability threshold. Using synthetic samples derived from SARS-CoV-2 and TuMV genomes, an evaluation of DVG-finder modes revealed that the meta-search mode was more sensitive than the ViReMa-a algorithm, whereas all modes were more accurate and F1 scores were better than the DI-tector algorithm (Olmo-Uceda et al., 2022).

Overall, DVG-finder incorporates the two most prevalent DVG detection algorithms and enhances their performance by ML, thus facilitating more efficient and precise identification and analysis of DVGs.

## 3 Primary types and generation mechanisms of DVGs

Research into DVGs has been hampered the absence of technology that could identify and distinguish DIPs from parental viruses (Manzoni and López, 2018). High throughput technologies have facilitated comprehensive studies, enabling the detection of various types of DVGs in most RNA virus families (Brennan and Sun, 2024). Deletion, copy-back (cb), and snap-back (sb) are primary types of DVGs (Vignuzzi and López, 2019). All three types can execute a full replication cycle with the help of parental viruses (Genoyer and López, 2019). The characteristics of these DVGs and how they are generated are described below.

### 3.1 Characteristics and generation of deletion DVGs

Deletion DVGs are prevalent in positive-strand RNA viruses such as alphaviruses, flaviviruses, picornaviruses, and coronaviruses (Li and Aaskov, 2014; Poirier et al., 2015; Liao et al., 2014; McLaren and Holland, 1974). The key characteristics are large internal deletions in critical genomic sequences, and preserved promoter sequences and cis-acting elements that play essential roles in replication and packaging (Li and Aaskov, 2014; Duhaut and Dimmock, 2000). Internal segments of the viral genome are missing from deletion DVGs. The DVGs are thought to be generated during viral genome replication as RNA dependent RNA polymerase (RdRp) pauses upon encountering secondary structures or lesion sites, then detaches from the template strand and reinitiates synthesis at new positions on the same or another template strand, producing daughter strands without internal regions. The production of deletion DVGs is considered to result from a replication-associated mechanism, during which RdRp transfers from a donor, to an acceptor template, while maintaining its connection to the nascent strand (Perrault and Semler, 1979).

### 3.2 Characteristics and generation process of copy-back DVGs

Non-segmented negative-strand RNA viruses such as paramyxovirus, respiratory syncytial virus, and filovirus frequently harbor cbDVGs that contain structurally rearranged genomes that have reverse complementation at the 5′ and 3′ ends; this configuration theoretically produces a long stem-loop structure (Perrault and Leavitt, 1978; Re et al., 1983; Kolakofsky, 1976). The mechanism underlying the generation of cbDVGs remain obscure. A leading hypothesis proposes that cbDVGs form during viral genome replication through a process in which the RdRp disengages from the parental template strand, reattaches to the daughter strand, uses it as a template, and extends synthesis from the 5′ end. The RdRp of negative-sense RNA viruses lacks proofreading activity, facilitating template switching. Additionally, high multiplicity of infection (MOI) or rapid replication increases RdRp error rates, thereby promoting the generation of cbDVGs (Lazzarini et al., 1981; Perrault, 1981).

### 3.3 Characteristics and generation process of snap-back DVGs

The characteristics and production process of sbDVGs are analogous to those of cbDVGs, with the distinction that their complementary double-stranded regions encompass almost the entire genome, and contain only one non-complementary nucleotide (Nichol et al., 1984).

## 4 Functions and mechanisms of DVGs

The DVGs can impede parental virus replication, induce interferon production, stimulate immune responses, and extend the duration of viral infection. These mechanisms are discussed below.

### 4.1 Inhibition of viral replication

Both DVGs and DIPs can directly interfere with the replication of standard viruses. Because the genome sequence in DIPs is shorter than that of standard viral genomes, they are smaller than intact virus particles (Li and Aaskov, 2014; Von Magnus, 1954; Calain and Roux, 1988; Treuhaft and Beem, 1982; Girgis et al., 2022; Huang et al., 1966). The shorter sequences of deletion DVGs and cbDVGs is thought to allow for stronger replication dynamics of the DIP genome (Huang and Baltimore, 1970). Two trailer promoter sequences characterize cbDVGs. The trailer promoter sequence in negative-strand RNA viruses is responsible for cbDVG formation and is more conducive to genome replication than the conventional leader promoter, thus granting cbDVGs a replication edge. In addition, DVGs in some species of viral DIPs enhance polymerase binding to nuclear proteins through mutations in promoter sequences, further augmenting their replication efficiency (Calain and Roux, 1995; Kolakofsky, 1979; Rao and Huang, 1982). Consequently, the DIP genome exploits the replication machinery that restricts access to proteins essential for standard virus replication, which results in a lower yield of the standard virus (Lazzarini et al., 1981; Perrault, 1981; Portner and Kingsbury, 1971; Giachetti and Holland, 1989). By competing for essential structural proteins necessary for packaging, DVGs can restrict the synthesis of standard viruses (Brennan and Sun, 2024). In conclusion, DVGs harboring cis-acting elements essential for genome replication and virion packaging can disrupt standard virus replication by competing for scarce polymerases, critical viral proteins, and other cellular resources in co-infected cells.

Elevated DVG levels can also decrease the synthesis of functional template mRNAs available for viral replication, thereby indirectly reducing the intracellular accumulation of viral proteins. The phenomenon occurs because deletion DVGs do not have essential coding regions for specific mRNAs, and duplication-derived DVGs cannot encode functional mRNAs (Brennan and Sun, 2024). Concurrently, accumulated DVGs in cells can stimulate PRRs, triggering a signaling cascade that enhances the expression of IFN and IFN-stimulated genes. As a result, the replication of standard viruses or the formation of standard virus particles is suppressed. For example, cells containing abundant DVGs, especially cbDVGs, can recognize and trigger signaling by mitochondrial antiviral signaling protein (MAVS) through RIG-I-like receptors. This leads to upregulated IFN types I and III signaling pathways and disrupted standard virus formation (Brennan and Sun, 2024; Genoyer and López, 2019; Manzoni and López, 2018).

### 4.2 Activation of innate immunity

Deletion DVGs, cbDVGs, and sbDVGs all elicit robust IFN responses. Specifically, cbDVGs activate the RIG-I-like receptor (RLR) pathway, which leads to the induction of pro-inflammatory cytokines such as interleukin (IL)-6, tumor necrosis factor-alpha (TNF-*α*), and IL-1β, thus promoting responses to IFN types I and III (Johnston, 1981; Tapia et al., 2013; Sun et al., 2015; Marcus and Gaccione, 1989; Strähle et al., 2007). This is attributed to the fact that unlike the standard viral genome, the specific 5′ end diphosphorylated or triphosphorylated blunt-ended double-stranded RNA structure of cbDVGs can theoretically form RIG-I ligands to bind with RLR. The innate immune response stimulated by cbDVGs boosts antigen presentation and concurrently drives dendritic cell maturation.

Deletion DVGs derived from DIPs associated with influenza, dengue, and Sindbis viruses induce the IFN response (Fuller and Marcus, 1980; Lin et al., 2022; Pelz et al., 2023). Deletion DVGs in poliovirus induce a powerful IFN response (Xiao et al., 2021). The abundance of DVGs and the magnitude of the IFN response significantly and positively correlated during the COVID pandemic (Zhou et al., 2023). Deletion DVGs produce PRRs such as RLRs that are activated during the replication of DVG or their double-stranded RNA intermediates, thus initiating the IFN pathway and resulting in the expression of IFN-stimulated genes (ISGs) and IFN generation. Meanwhile, recent studies reveal that some coronavirus DVGs encode cryptic proteins capable of suppressing RIG-I signaling—challenging existing paradigms of DVG-host interactions (Hsu et al., 2025). However, the specific sequences or secondary structures within deletion DVGs that could be recognized by RLRs have not been identified.

### 4.3 Association with viral persistence

Although DVGs and DIPs interfere with the generation of standard viruses, the quantity of these generated viruses conversely influences DVGs and DIPs. Standard virus infection of a single cell results in neighboring cells becoming infected. As the amount of standard viruses increase in a single cell, DVGs/DIPs are synthesized *de novo*. Thereafter, DIPs replicate dependently on standard viruses and hinder their replication (as described in section 2.2). This leads to DIP accumulation and decreased standard virus titers. A reduction in the titer of standard viruses within a cell population ultimately causes a significant decrease in the amount of cells simultaneously infected by standard viruses and DIPs. As DIPs require standard viruses for replication and packaging, cells infected exclusively by DIPs cannot propagate, which causes a decline in DIP titers. In contrast, cells infected with standard viruses can only produce standard virus particles, which increases titers. When the titer of standard viruses increases, DVGs and DIPs will recommence generation, initiating a new cycle. This is referred to as the DIP and standard virus asynchronous cycle or the Von Magnus effect (Huang and Baltimore, 1970) that renders persistent and cyclical viral infection.

In addition, the composition of viral genome species is heterogenous. Some cells are enriched with DVGs, whereas others are enriched in canonical viral genomes. Mitochondrial antiviral signaling proteins are activated by cells with abundant DVGs, resulting in the production of IFN and other proinflammatory cytokines, such as TNF, which has dual functions during infection, contingent upon engaged signaling pathways. Specifically, TNF acts as a proinflammatory and pro-apoptotic agent when signaling through the TNFR1 pathway, but functions as a pro-survival factor *via* the TNFR2 pathway. Activating the TNFR1 pathway promotes the apoptosis of cells enriched with canonical viral genomes. Conversely, the TNFR2 pathway, along with downstream signaling molecules such as TNF receptor-associated factor 1 (TRAF1), prolongs the survival of cells with elevated DVG levels, which extends persistent infections (Xu et al., 2017). Collectively, DVGs can facilitate the persistent infection of viruses.

**Figure 2:**
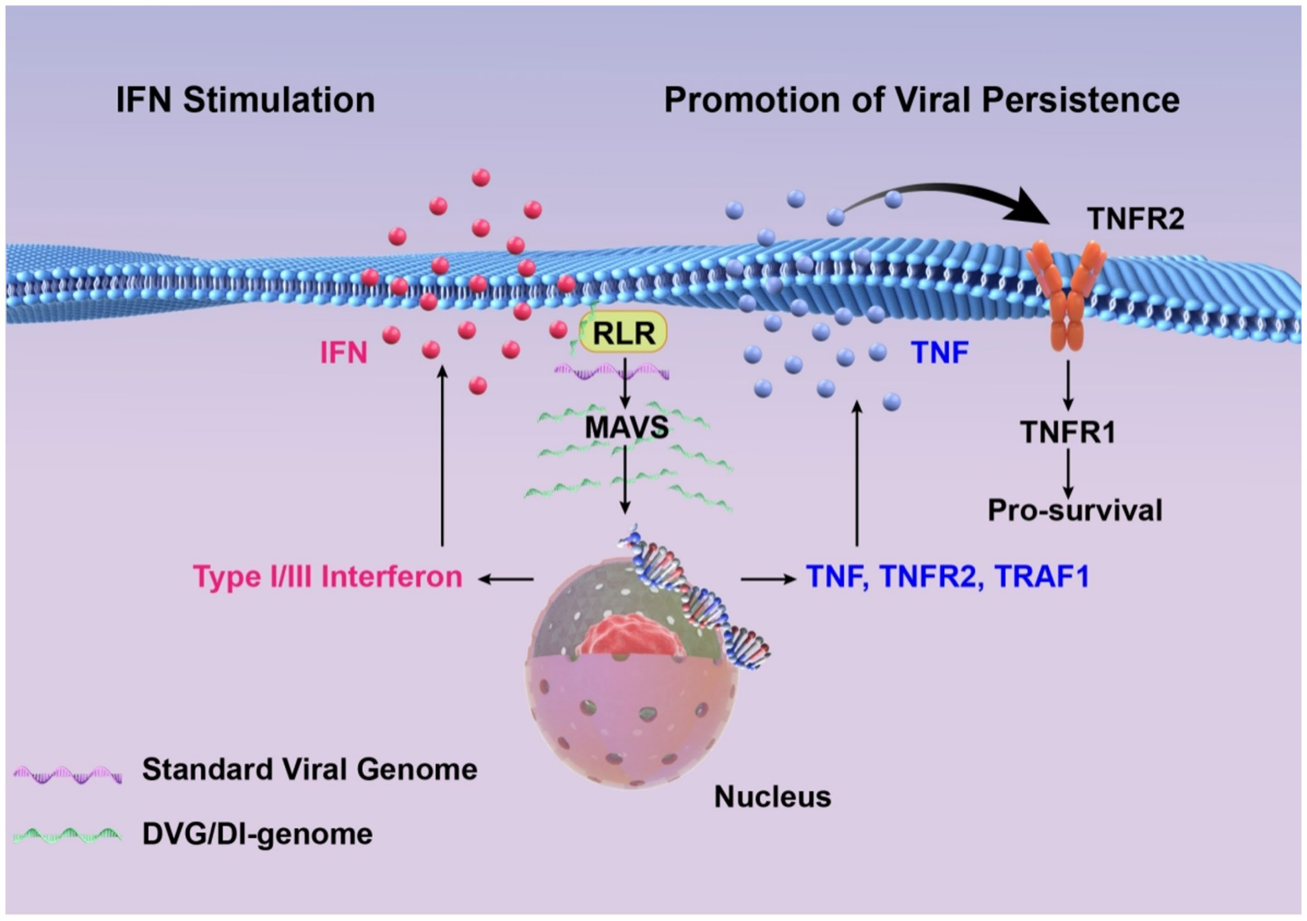
High concentrations of DVGs significantly enhance type I/III interferon signaling by detecting and activating RIG-I-like receptors and MAVS signaling pathways. DVGs-rich cells activate the cell survival pathway by up-regulating TNF/TNFR2/TRAF1, and combine with a small amount of standard viral genome to maintain a persistent infection state.

Overall, the inhibition of standard virus replication is closely associated with the role of IFNs. Furthermore, the establishment of persistent infections is related to the replication of standard viruses and the production of IFN and other cytokines (Figure 2). Therefore, the interference of standard virus replication with DVGs and DIPs, the induction of IFN generation, immune response activation, and prolonged infection cycles are not mutually independent processes, but they notably correlate.

## 5 Progress in clinical applications of DVGs

Because of their capacity to disrupt normal viral replication and trigger production, DVGs are considered attractive options for antiviral treatments (Genoyer and López, 2019; Stegmann et al., 2022). Live attenuated vaccines frequently contain DIPs, theoretically supporting the notion that DVGs could substitute for conventional vaccine adjuvants. In addition to these two primary areas, DVGs might play special roles in enhancing clinical management (Felt et al., 2021) and in developing anti-tumor medications (Lu et al., 2021; Yang et al., 2019).

### 5.1 Antiviral medication therapy

After host viral infection, DIPs are naturally generated and impact infection through mechanisms such as interfering with viral replication and stimulating immune responses (Rezelj et al., 2018; Dimmock and Easton, 2014; Dimmock et al., 1986; Ziegler and Botten, 2020). Consequently, DIPs are promising antiviral therapeutic agents (Dimmock and Easton, 2014; Doyle and Holland, 1973; Chambers and Webster, 1991; Nelson and Perelson, 1995). Furthermore, various therapeutic interfering particles (TIPs) have been created (Metzger et al., 2011; Weinberger et al., 2003) that has encouraged the application of DVGs to clinical settings. In fact, DIPs and TIPs based on RNA viruses such as SARS-CoV-2, flavivirus, and Influenza A Virus (IAV), have advanced to the animal experimental stage [a systematic review of IAV DIPs has been published (Wu et al., 2022)]. They typically offer benefits including low susceptibility to drug resistance and broad-spectrum antiviral activity, which support further investigation and the creation of novel antiviral medications (Table 2).

**Table 2:** A Summary of DVGs applications in antiviral therapy from 2019 to 2024.

Name	Source	Mode of action	Advantage	Disadvantage
eTIP1 (Xiao et al., 2021)	Capsid coding region of poliovirus	Triggers interferon response to exert broad-spectrum anti-infection effects; stimulates production of neutralizing antibodies to provide immune protection	Safety features; low drug resistance	Might induce cytokine storm (Fajgenbaum and June, 2020; Findlay et al., 2015; Yuan et al., 2021)
Lipid nanoparticles TIPs (Chaturvedi et al., 2021; Chaturvedi et al., 2022)	Subgenomic RNA of SARS-CoV-2	Reduce the viral load; Down-regulate relevant genes to inhibit the pro-inflammatory immune response; shorten the nasal shedding time of SARS-CoV-2	A more long-lasting protective effect; low drug resistance	It is still unclear whether its generation and dissemination are spontaneous; it is unable to completely block the transmission of the virus
DIPs (Lin et al., 2022)	Purified DENV-2	Inhibit viral replication; Activate the type I interferon response; may quickly trigger the innate immune response of the host	Inhibit the replication of Dengue virus, Zika virus, Yellow fever virus, Respiratory syncytial virus and the Omicron variant of SARS-CoV-2	The impact on the risk of antibody-dependent enhancement remains unclear
IAV DIPs (Pelz et al., 2021; Rand et al., 2021; Huo et al., 2020)	IAV	Induce an innate immune response that signaled through janus kinase/signal transducer and activator of transcription (JAK/STAT); increase the production of type I and type III interferons	Broad-spectrum antiviral activity, completely inhibit the replication of SARS-CoV-2; rapid action; low drug resistance	There are difficulties in production and purification
OP7 (Hein et al., 2021; Kupke et al., 2019)	The genomic vRNA of S7 of IAV that harbors 37 point mutations	Significantly reduce the viral titer; stimulate the innate immune response	Successfully fight against IAV infection	There may be differences in purity, activity and other aspects among different batches of OP7
DI244 (Bdeir et al., 2019; Hein et al., 2021)	Pure cloned IAV free from the contamination of Standard Virus	Interfere with viral replication; induce interferon production	Broad-spectrum antiviral activity; rapid action; low drug resistance; more convenient for production	The production quality cannot be assured
NiV DIPs (Welch et al., 2020)	NiV	Reduce the viral titer; compete with the parental viral genome for the viral protein pool and some host cell factors, thus interfering with replication and assembly	Directly reduce the replication of NiV *in vitro*; provide immediate protection	The design and production remain to be optimized; there is a potential risk of drug resistance
CHIKV DVGs (Levi et al., 2021)	CHIKV	Compete with the wild-type virus for resources needed for replication, thereby inhibiting replication; may interfere with the synthesis of viral structural proteins, thus affecting the assembly process	Broad-spectrum antiviral activity; Pre-injection can inhibit the spread of the virus within mosquitoes; has potential immune-stimulating ability.	The frequency of its occurrence in mosquitoes is minimal; it is difficult to separating it from the wild-type virus; it is difficult to choose a suitable *in vivo* model to assess its effectiveness
Zika virus TIPs (Rezelj et al., 2021)	Screen for DVG-A after serial passage of Zika virus at a high MOI, and then package them into TIPs	Competing with the wild-type virus for essential viral proteins required for replication and packaging, as well as relying on an intact RNA interference pathway to suppress replication	Inhibit viral infection and transmission, which may avoid the occurrence of complications; reduce the transmission of the virus within mosquitoes	The effectiveness in different situations needs to be further demonstrated; long-term use may induce immune problems
DIPs without infectious DENV-2 (Li et al., 2021)	DENV - 2	Inhibit viral replication; activate the interferon response	Broad-spectrum antiviral activity; absence of transmissible viral contamination risk	The mechanism of action remains to be elucidated; *in vivo* investigations are currently limited
RSV cbDVGs (Felt et al., 2021)	RSV	Interfere with viral replication; induce the expression of interferons and interferon-stimulated genes	Predict the severity of RSV infection; guide clinical management and improve the prognosis	Easily affected by other factors; there are limitations in the detection technology

DENV-2 is Dengue virus serotype 2, IAV is Influenza A virus, vRNA is viral RNA, S7 is segment 7, NiV is Nipah virus, MOI is multiplicity of infection.

Most synthetic DIPs and TIPs are deletion-type, meaning that their genomes lack nucleotide sequences and require parental virus support for dispersal. A small proportion of these are cb types that are usually found in paramyxoviruses (see Section 2.1.2). Recent studies have identified a new class of DIPs (Kupke et al., 2019). In contrast to the traditional deletion types, these DIPs have point mutations in their genomes.

Both DIPs and TIPs conventionally co-infect facilitated by the parental virus, and they target the same infected cells and tissues. This process disrupts replication of the parental virus, with significant specificity. Moreover, DIPs possess broad-spectrum activity because they compete with other viruses for cell surface receptors, intracellular enzymes, nucleotides, and other resources necessary for replication, even though they do not mediate heterologous viral interference (Huang and Baltimore, 1970). This has been found when DIPs (Li et al., 2021) act in the absence of infectious Dengue virus 2 (DENV-2). DIPs can cause host cells to create neutralizing antibodies and IFN responses, which lowers viral titers, decreases disease severity, and shields the host from further viral infections. For instance, IAV DIPs (Rand et al., 2021), by activating responses of IFN types I and III, can suppress IAV replication, while alleviating SARS-CoV-2 infection.

The emergence of the novel coronavirus during 2019 significantly influenced daily living and global productivity. Despite notable progress in vaccines (Zasada et al., 2023; Wang et al., 2024), and therapies (Li et al., 2020; Su et al., 2020; Aanouz et al., 2021), high viral mutation rates (Focosi and Maggi, 2021; Ghildiyal et al., 2024; Planas et al., 2021; Cao et al., 2022; Garcia-Beltran et al., 2021) and drug resistance (Iketani et al., 2023; Duan et al., 2023) still hinder treatment effectiveness. Therefore, improving the effects of medications are crucial. In this context, antiviral strategies based on DIPs have been investigated to treat COVID-19. An antiviral eTIP1 created by deleting the coding region of the poliovirus capsid stimulates the immune system and initiates the IFN response in mice (Xiao et al., 2021). The approach also offers decreased drug resistance and increased safety features. Furthermore, inflammation, lung edema, and pulmonary viral loads are decreased in hamsters administered with intranasal TIPs (Chaturvedi et al., 2021). At lower dosages, the antiviral activity of TIPs resembles that of small-molecule antiviral medications and monoclonal antibodies. This represents a significant advantage of TIPs in clinical research. Defective viral genomes are frequently generated during SARS-CoV-2 infection, and they contribute to prolonged viral replication and the induction of responses to IFN types I and III. Overall, these results lay the groundwork for the subsequent development of antiviral medications and related vaccines against SARS-CoV-2 (Zhou et al., 2023).

Antiviral therapy based on DVGs offers significant advantages over conventional treatments as they can specifically spread alongside their parent virus, which makes it easier for them to exert a therapeutic effect. In addition, this strategy is less likely to cause drug resistance and broad-spectrum antiviral action. However, several obstacles associated with safety, production, and quality control must be overcome before DVGs could be applied to clinical treatment.

### 5.2 Vaccine adjuvants

**Figure 3:**
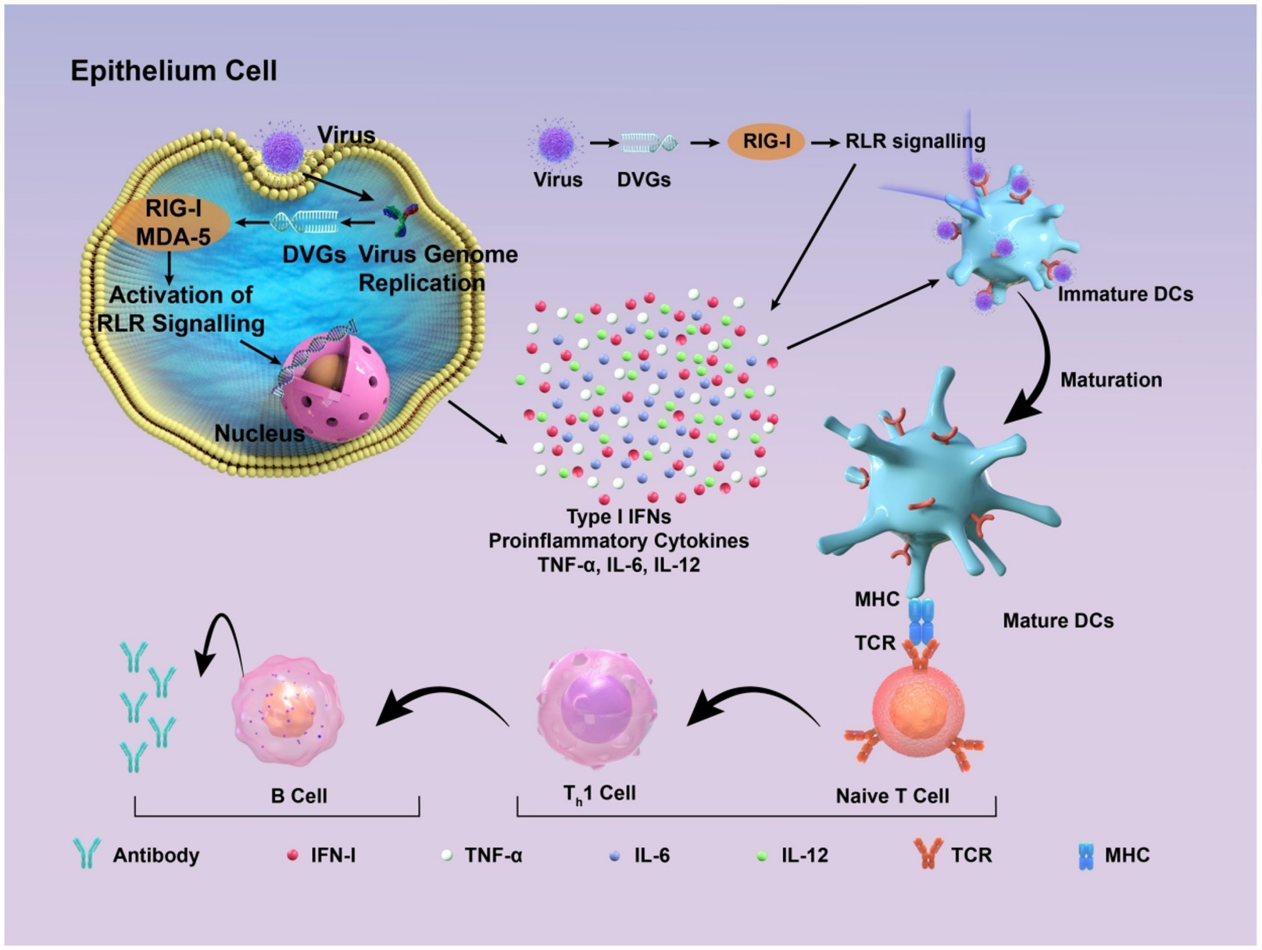
Schematic diagram of how DIPs activate host immune defense. DVGs are present in DIPs. Retinoic acid-inducible gene I (RIG-I) and melanoma differentiation-associated protein 5 (MDA-5) recognize copyback DVGs and then trigger the creation of interferons (IFNs) and other cytokines, which supports the activation of the innate immune response. These factors contribute to the maturation of dendritic cells, thus contributing to the adaptive immune response. Subsequently, DVGs can enhance the recognition between naive T cells and mature dendritic cells, promoting the cell-mediated immune process mediated by type I IFN signaling. This, in turn, stimulates B cells to produce corresponding antibodies, thereby exerting the function of humoral immunity.

In addition to aiding the development of antiviral medications, DVGs might be useful as adjuvants that increase the effectiveness of vaccines via several mechanisms, such as PRR stimulation (Reijers et al., 2022). Defective interfering particles are abundant in current live-attenuated vaccines, including those for poliovirus (McLaren and Holland, 1974), measles (Bellocq et al., 1990), and influenza (Gould et al., 2017) viruses. They have several benefits, such as safety, immunogenicity, and the ability to naturally permeate cells and target multiple PRRs. Specifically, they can induce host immune defense (Vasou et al., 2017) (Figure 3).

RNA oligonucleotides and 268 nt oligonucleotides derived from the DVGs of Sendai virus, are intriguing new immune-inducing adjuvants because of their low cost, high stability, favorable sequences, and potential for large-scale manufacture (Fisher et al., 2018; Mercado-López et al., 2013). Recent studies demonstrate that defective interfering RNAs (DI RNAs) from Sendai virus and influenza virus, when employed as vaccine adjuvants, can promote Th1-type immune responses, shift IgG subtypes toward IgG2b, and induce Th17-type immune responses. Moreover, lipid nanoparticles (LNPs) or nanoemulsions (NE) can work in concert with these adjuvants to improve immune responses. Critically, in murine models challenged with homologous influenza viruses, vaccines adjuvanted with such DVG-RNAs significantly reduce weight loss and suppress pulmonary viral replication—protective outcomes strongly correlated with augmented immune responses (Jangra et al., 2024; Wong et al., 2021).

The live-attenuated recombinant vaccine candidate ML29 comprising Mopeia and Lassa viruses, is a promising treatment for Lassa fever (Hallam et al., 2018). Attenuation is high in animal models when DIPs are enriched in ML29. In particular, CBA/J mice, STAT-1^−/−^ mice, and Hartley guinea pigs had no signs or symptoms, serious illness or abnormalities, indicating that vaccines containing DIPs could improve safety and immunogenicity and aid the induction of wider cross-protection (Johnson et al., 2021).

Although DVGs offer advantages over traditional vaccine adjuvants, they might also interfere with vaccine production (Zinnecker et al., 2024). For example, they can raise the complexity and impurity levels of the production process and impact the stability of continuous virus generation. Moreover, suitable materials and technological procedures must be developed to prevent DIPs from degrading quickly *in vivo* and to allow for precise targeting of immune cells.

DVGs possess significant potential for application in disease diagnosis and prognosis, although current research in this domain remains limited in scope. The content of DVGs is linked to the severity of the disease (Vasilijevic et al., 2017; Penn et al., 2022), which could help anticipate how the condition will progress. According to a study on respiratory syncytial virus (RSV) DVGs (Felt et al., 2021), identifying DVGs in hospitalized and non-hospitalized patients can help physicians create customized diagnosis and treatment plans, making it easier to identify patients who require admission to the intensive care unit (ICU). Thus, DVGs have the potential to serve as a biomarker for ICU admission.

### 5.3 Clinical applications: from RNA viruses to DNA viruses

It should be noted that although RNA viruses have received the majority of attention in DVG research, dsDNA viruses—especially herpesviruses—should also be considered. Two types of HSV-1 DVGs were identified decades ago. Researchers have been delving deeply into the properties and processes of HSV-1 DVGs development in recent years, uncovering the sequence-driven and cell-specific mechanisms that underlie these formations (Shitrit et al., 2023). This gives us a foundation to comprehend their function in viral infection and evolution. HSV-1-derived amplicon vectors and non-replicating genomic vectors have also demonstrated promise in gene therapy (Le Hars et al., 2025; Ingusci et al., 2025). Apart from herpesviruses, analysis of urine samples from immunosuppressed patients revealed the presence of DVGs in both BK and JC polyomaviruses, with persistence observed throughout the infection course. This study provides the first confirmation of DVG prevalence in clinical DNA virus samples, expanding our understanding of their role in clinical virology (Addetia et al., 2021).

## 6 Conclusion

Since the discovery of DVGs studies have encountered significant challenges. Recent technological advancements have enabled the detection and sequencing of DVGs in experimental and natural viral infections, thus deepening understanding of the mechanisms of their production and function, and expanding the diversity of the DVG family. Moreover, DVG-based antiviral therapies might offer advantages over traditional approaches. They co-transmit with standard viruses and have broad-spectrum antiviral activity. Furthermore, protective effects mediated by DVGs are rapid, potent, and durable, with a lower likelihood of inducing drug resistance. However, the application of DVGs to therapeutic settings still faces several challenges. The design of effective DIPs is complex, and computational methods have difficulty identifying optimal antiviral candidates (Pelz et al., 2021). Owing to transmission bottlenecks, TIPs cannot be effectively transmitted between hosts (Chaturvedi et al., 2022), thus requiring more advanced production techniques. Current research is limited to experimental animal models, and extending these findings to human trials remains a significant challenge because of inherent differences in data sources between human and other animal models (Li et al., 2024; Chaturvedi et al., 2021). Moreover, contamination due to genetic recombination during the preparation of DIPs might be a potential concern (Barone et al., 2020). Given the widespread incidence of DVGs in live attenuated vaccines, intensifying research on these vaccines is imperative to gain new insights into the mechanisms of action of DVGs. This review primarily focuses on RNA viruses. However, almost all families of viruses harbor DVGs, but much remains to be discovered. Therefore, further investigation is required to expand the depth of knowledge. We anticipate that DVGs will be clinically applied to alleviate and treat various human diseases after questions are answered and problems are addressed.
