# Correction: Identification, functional analysis, and clinical applications of defective viral genomes

**DOI:** 10.3389/fmicb.2025.1670478

**Published:** 2025-08-11

**Authors:** Xiaowei Yan, Yitong Pan, Peiying Li, Li Zhu, Jianhai Yu, Chenguang Shen, Bao Zhang, Wei Zhao

**Affiliations:** ^1^BSL-3 Laboratory (Guangdong), Guangdong Provincial Key Laboratory of Tropical Disease Research, Ministry of Education Key Laboratory of Infectious Diseases Research in South China, School of Public Health, Southern Medical University, Guangzhou, China; ^2^School of Biomedical Engineering, Southern Medical University, Guangzhou, China

**Keywords:** interfering particle, defective viral genome, identification, function, application

In the published article, there was an error in positions of figures as published. Due to an oversight during the proofreading stage, Figure 2 and Figure 3 were swapped in the published version, resulting in a mismatch between the figure and their actual content. The corrected positions of figures and captions appear below.

**Figure 2:**
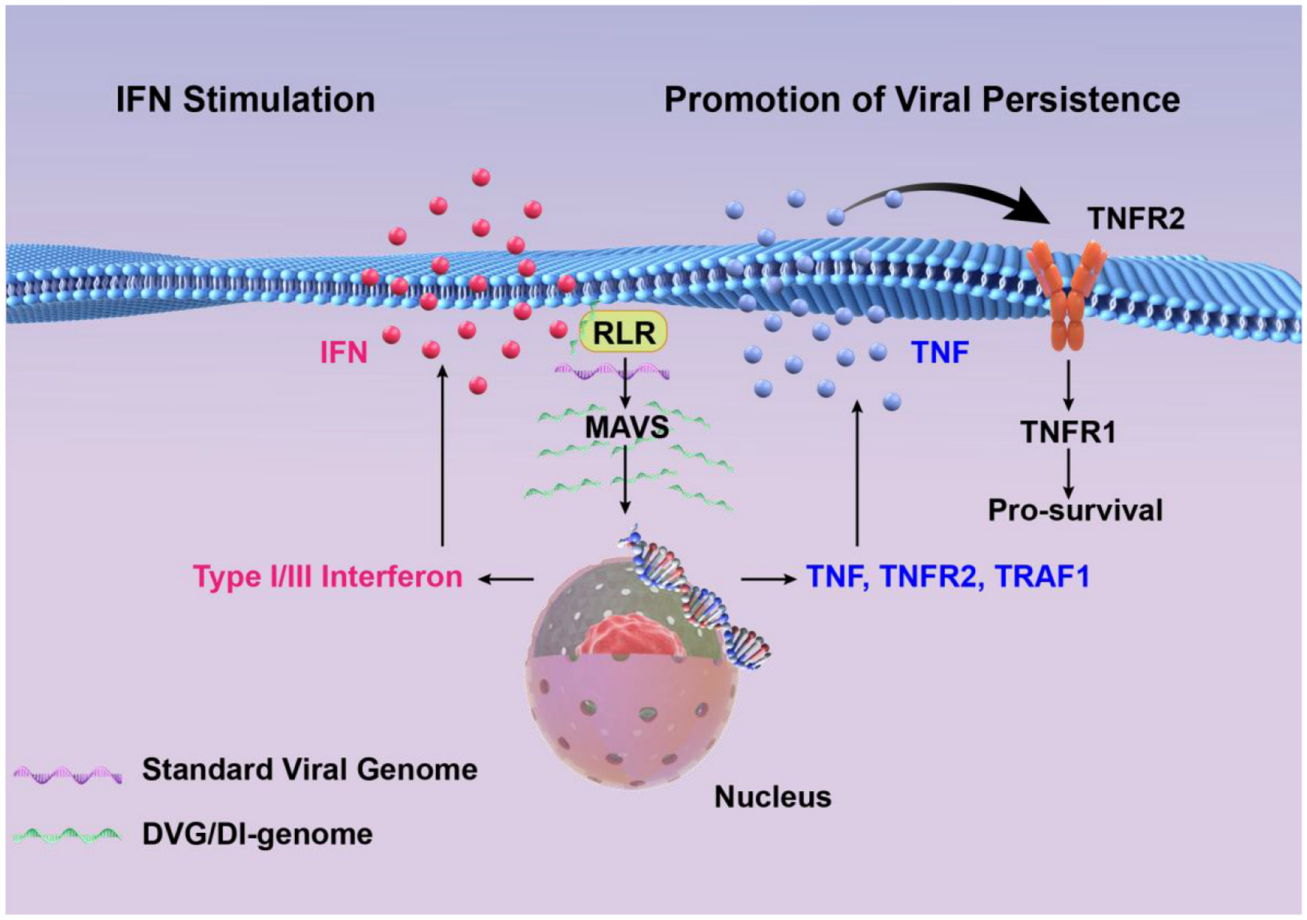
High concentrations of DVGs significantly enhance type I/III interferon signaling by detecting and activating RIG-I-like receptors and MAVS signaling pathways. DVGs-rich cells activate the cell survival pathway by up-regulating TNF/TNFR2/TRAF1, and combine with a small amount of standard viral genome to maintain a persistent infection state.

**Figure 3:**
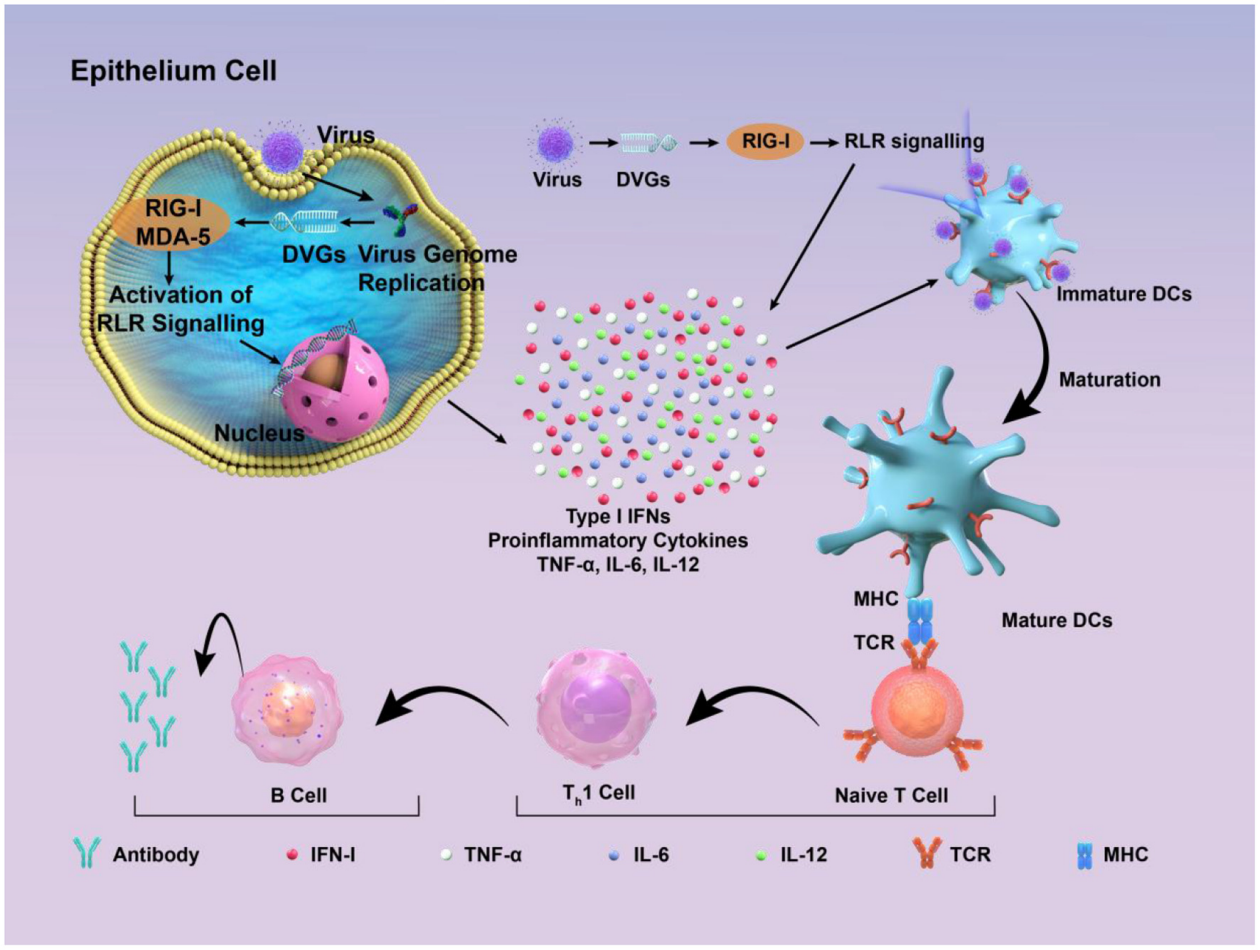
Schematic diagram of how DIPs activate host immune defense. DVGs are present in DIPs. Retinoic acid-inducible gene I (RIG-I) and melanoma differentiation-associated protein 5 (MDA-5) recognize copyback DVGs and then trigger the creation of interferons (IFNs) and other cytokines, which supports the activation of the innate immune response. These factors contribute to the maturation of dendritic cells, thus contributing to the adaptive immune response. Subsequently, DVGs can enhance the recognition between naive T cells and mature dendritic cells, promoting the cell-mediated immune process mediated by type I IFN signaling. This, in turn, stimulates B cells to produce corresponding antibodies, thereby exerting the function of humoral immunity.

The original article has been updated.

